# Effect of Probiotics Containing *Lactobacillus plantarum* on Blood Lipids: Systematic Review, Meta-Analysis, and Network Pharmacological Analysis

**DOI:** 10.3390/foods14193300

**Published:** 2025-09-23

**Authors:** Jinshi Zuo, Dan Huang, Jie Liu, Zidan Wang, Yuerong Ren, Yang Su, Yuxia Ma

**Affiliations:** 1 Hebei Key Laboratory of Environment and Human Health, Department of Nutrition and Food Hygiene, School of Public Health, Hebei Medical University, Shijiazhuang 050017, China; zuojinshi2022@163.com (J.Z.); liujie@stu.hebmu.edu.cn (J.L.); wzdguodanpi@163.com (Z.W.); 17526937248@163.com (Y.R.); 2 Undergraduate of College of Public Health, Hebei Medical University, Shijiazhuang 050017, China; m18290085874@163.com (D.H.); suyangsssyyy@163.com (Y.S.)

**Keywords:** probiotic, *L. plantarum*, blood lipids, meta-analysis, network pharmacology analysis

## Abstract

Background: Cardiovascular diseases, driven significantly by dyslipidemia, remain a leading global mortality risk. Emerging evidence indicates that *Lactobacillus plantarum* (*L. plantarum*), which is a probiotic commonly used in a variety of food products, may contribute to the regulation of blood lipids, although prior studies report inconsistent efficacy and lack mechanistic clarity. This study aimed to evaluate the effects of *L. plantarum* supplementation on blood lipid profiles and explore its potential mechanisms through a systematic review, meta-analysis, and network pharmacology. Methods: We performed a comprehensive literature search across PubMed, Cochrane Library, EMBASE, and other databases. Meta-analysis was performed using random-effects models to assess changes in total cholesterol (TC), triglycerides (TG), high-density lipoprotein cholesterol (HDL-C), and low-density lipoprotein cholesterol (LDL-C). Network pharmacology was employed to predict molecular targets and pathways. Results: Twenty-six randomized controlled trials (RCTS) involving 2104 participants were included. *L. plantarum* supplementation significantly reduced TC (SMD: −0.233; 95% CI: −0.458, −0.008; *p* = 0.042), TG (SMD: −0.227; 95% CI: −0.432, −0.021; *p* = 0.030), and LDL-C (SMD: −0.251; 95% CI: −0.477, −0.025; *p* = 0.029), but not HDL-C. Subgroup analyses revealed greater efficacy with interventions lasting >8 weeks and single-strain formulations. Network pharmacology analysis highlighted IL-17/TNF signaling pathway, bile secretion, and other pathways as key mechanisms and targets such as PPARG and MMP9 as key targets. Conclusions: *L. plantarum* demonstrates significant lipid-lowering effects, particularly for TC, TG, and LDL-C, with sustained use and single-strain formulations yielding optimal outcomes. Mechanistically, it may modulate inflammation, oxidative stress, and lipid metabolism. These findings can support the development of a functional food and dietary supplement using *L. plantarum* to assist in the treatment of hyperlipidemia, though heterogeneity and strain-specific effects warrant further investigation.

## 1. Introduction

Cardiovascular disease (CVD) ranks as the primary cause of mortality across most developed and developing nations. Although several reports have demonstrated a decline in the CVD-associated mortality rate [1,2], it is projected that by 2030, nearly 23.3 million individuals will succumb to CVD [3]. Dyslipidemia, a key risk determinant for CVD, is characterized by perturbations in the levels of total cholesterol (TC), low-density lipoprotein cholesterol (LDL-C), high-density lipoprotein cholesterol (HDL-C), and triglycerides (TG) [4]. Hyperlipidemia, the most prevalent form of dyslipidemia, is a well-established feature of metabolic disorders including metabolic syndrome, obesity, and CVD [4,5]. Its pathogenesis is rooted in disturbances in lipid metabolism [6,7,8,9]. Numerous studies have demonstrated that elevated lipid levels can inflict significant damage on the vascular system and multiple organs, contributing to conditions such as obesity and hyperglycemia [10,11]. Consequently, the effective prevention and management of hyperlipidemia and cardiovascular diseases are crucial for enhancing public health outcomes.

Emerging research indicates that probiotic supplementation has emerged as an innovative therapeutic modality for cardiovascular disease management [12]. *Lactobacillus plantarum* (*L. plantarum*) is widely distributed in nature and is commonly found in a diverse range of fermented foods as well as within the gastrointestinal tracts of animals. It is a preeminent probiotic strain, has demonstrated promising therapeutic efficacy, including immune system stimulation, gastrointestinal function regulation, and serum cholesterol reduction [13,14]. Recently, there has been interest in *L. plantarum*’s function in regulating lipid metabolism [15,16,17]. Animal studies have clearly demonstrated the salutary effects of *L. plantarum* on blood lipid regulation [18,19,20]. Additionally, several randomized controlled trials (RCTs) have investigated the impact of *L. plantarum* on blood lipid profiles in both normolipidemic and moderately hyperlipidemic human populations [21,22]. Although some RCTs in humans have reported that *L. plantarum* effectively regulates blood lipids [22,23,24,25], others have failed to observe any significant effect [26,27].

Previous meta-analyses of RCTS in humans summarized the efficacy of *L. plantarum*-containing probiotics in regulating blood pressure and the role of *L. plantarum* in reducing obesity and inflammation [28,29]. However, up to now, the relationship between *L. plantarum* intake and lipid profile is not comprehensive. Therefore, we conducted a systematic review and meta-analysis on the basis of a literature search and comprehensively analyzed the effect of *L. plantarum* supplementation on lipid profiles. In addition, we also used network pharmacology to predict the potential mechanism of lipid lowering by *L. plantarum*. In accordance with PRISMA guidelines, all relevant randomized controlled trials (RCTs) that met the predefined reporting criteria were included to evaluate the effects of *L. plantarum* supplementation on lipid profiles. The results of this study are of great significance for the development of functional foods and dietary supplements based on *L. plantarum*, in order to achieve lipid management.

## 2. Methods

This systematic review and meta-analysis were conducted in accordance with the Preferred Reporting Items for Systematic Reviews and Meta-Analyses (PRISMA) guidelines, and the review was registered a priori in the International Prospective Register of Systematic Reviews (PROSPERO; registration number: CRD42024608598).

### 2.1. Search Strategy

We performed a comprehensive literature search in PubMed, Cochrane Library, EMBASE, Web of Science, and Scopus, from inception to 13 October 2024. A comprehensive literature search was performed to identify all relevant studies investigating the effects of *L. plantarum* supplementation on lipid profiles, with no restrictions applied to publication language or time frame. The search strategy included the following Medical Subject Headings (MeSH) and non-MeSH terms: “*Lactobacillus plantarum*” OR “*L. plantarum*.” AND “cholesterol” OR “total cholesterol’ OR “Lipoproteins, HDL’ OR “Lipoproteins, LDL” OR “triglycerides” OR “triacylglycerol” OR “blood profile” OR “Cholesterol, HDL” OR “Cholesterol, LDL” OR “Cholesterol, VLDL”. All included studies were limited to RCTs. The specific search strategy string for each database is provided in the Supplementary Materials.

### 2.2. Study Selection and Eligibility Criteria

First, all relevant research articles were imported using EndNote X9 software (Clarivate, Philadelphia, PA, USA), and duplicate entries were systematically removed. Subsequently, two reviewers (HD and SY) independently screened the studies by examining titles, abstracts, and full texts when necessary. The PICO (Population, Intervention/Exposure, Comparison, Outcome) framework was adopted to circumscribe the scope of this review: Population: adults who had attained 18 years of age or older. Exposure: the application of *L. plantarum* by the study participants. Comparison: a comparative analysis between the group of participants exposed to *L. plantarum* and the non-exposed control group. Primary Outcome: alterations in the blood lipid parameters of the participants. The following criteria were established for study inclusion: (1) the research adhered to the randomized controlled trial (RCT) design; (2) the study focused on the evaluation of *L. plantarum* (including all subspecies), administered as either a single-strain or multi-strain dietary supplement; (3) both baseline and outcome data for the intervention and control groups were presented; (4) the duration of the treatment intervention was not less than two weeks. Conversely, studies were excluded if they met any of the following criteria: (1) the research involved non—human subjects; (2) studies employing non-RCT designs (e.g., retrospective studies, cohort studies, case–control studies, cross-sectional analyses) were excluded; (3) the investigation lacked a proper comparison with a control group; (4) the duration of the treatment intervention was less than two weeks. In the event of any discrepancies in the screening decisions between the two reviewers, a third reviewer (ZJS) was consulted to facilitate the resolution.

### 2.3. Data Extraction

Data extraction was performed using a standardized electronic form to collect the following details from each study: (1) first author, publication year, country, and sample size; (2) study methodology including design type, intervention duration, and dosage; (3) participant characteristics such as the total number, age, gender, and baseline lipid profiles. Throughout the data extraction process, whenever discrepancies or disagreements emerged between the two reviewers, these were resolved through a consultative process involving a third reviewer (ZJS). In accordance with standard methodological practice, we endeavored to acquire missing data by directly contacting the authors of the primary studies.

### 2.4. Quality Assessment of the Studie

The Cochrane Collaboration’s standardized methodological guidelines were rigorously employed to evaluate the risk of bias associated with the overall quality of the studies included in this review [30]. The assessment framework encompasses seven distinct domains for quality appraisal: (1) random sequence generation; (2) allocation concealment; (3) blinding of participants; (4) blinding of outcome assessment; (5) free of incomplete outcome; (6) free of selective reporting; (7) other bias. Each of these domains was systematically classified as presenting a low, high, or unclear risk of bias. During the quality assessment procedures, in the event of discrepancies between the two primary reviewers, a third reviewer was engaged to participate in the evaluation process. Subsequently, the entire author group conducted in depth discussions to resolve the issue and reach a consensus.

### 2.5. Network Pharmacological Analysis

The metabolites of *L. plantarum* were retrieved from the literature [31]. Based on Lipinski’s Rule of Three (Ro3), compounds were screened using the PubChem database (https://pubchem.ncbi.nlm.nih.gov/, accessed on 1 March 2025) with the following criteria: molecular weight ≤ 300, logP (octanol-water partition coefficient) ≤ 3, hydrogen bond donors ≤ 3, hydrogen bond acceptors ≤ 3, and rotatable bonds ≤ 3. The Isomeric SMILES identifiers of active components were recorded, and their SDF files were downloaded from PubChem. Target prediction was performed using Swiss Target Prediction (http://www.swisstargetprediction.ch/, accessed on 1 March 2025) and TargetNet (http://targetnet.scbdd.com/, accessed on 1 March 2025). Targets with Probability > 0 were selected. Disease targets related to hyperlipidemia were collected from the OMIM database (https://omim.org/) and GeneCards (https://www.genecards.org/; Relevance score > 10) using “hyperlipidemia” as the keyword. A Venn diagram was generated using the R.4.5.1 package to identify overlapping targets. The overlapping targets were imported into the STRING database (https://string-db.org/) to construct a PPI network. Cytoscape 3.7.2 with the CytoHubba plugin was employed to refine core targets using the Maximal Clique Centrality (MCC) algorithm. Gene Ontology (GO) and Kyoto Encyclopedia of Genes and Genomes (KEGG) pathway analyses were performed using the DAVID database (https://davidbioinformatics.nih.gov/, accessed on 1 March 2025). Significant terms and pathways were filtered with a *p*-value < 0.05.

### 2.6. Statistical Analysis

The meta-analysis was executed using STATA11 software and R.4.5.1. To comprehensively summarize the effective index of quantitative data, 95% confidence intervals (CIs) and standardized mean differences (SMDs) were applied. For studies presenting diverse intervention dosages and durations, we deliberately selected the maximum dosages and the longest durations for the outcomes.

To account for the heterogeneity across studies, a random-effects model was adopted in the meta-analysis, as per the approach proposed by Feng et al. [32]. The heterogeneity among studies was evaluated using I^2^ statistics. Specifically, heterogeneity was deemed statistically significant when the I^2^ value exceeded 50%.

Sensitivity analyses were conducted to recalculate the pooled effect estimates and heterogeneity following the exclusion of each individual trial. Studies that removed altered heterogeneity or effect significance were considered influential. In addition, subgroup analyses were explored to assess the heterogeneity caused by study duration (≤ 8 weeks and > 8 weeks) and the content of supplements used in the intervention (*L. plantarum* supplements or multi-strain supplements containing *L. plantarum*).

A meta-regression was conducted to investigate potential sources of heterogeneity among the included studies and to assess trends in lipid levels over time based on publication year. The following covariates were considered: sample size of the intervention group, geographic region, mean baseline TC level, mean baseline age, and the dose–response association with mean baseline BMI. Additionally, we performed a comprehensive dose–response meta-analysis. We utilized the restricted cubic spline (RCS) model with three knots to explore potential nonlinear relationships, as well as a simple linear meta-regression model, with daily dosage (ln-transformed CFU/d) as the independent variable.

## 3. Results

### 3.1. Search Results

**Figure 1:**
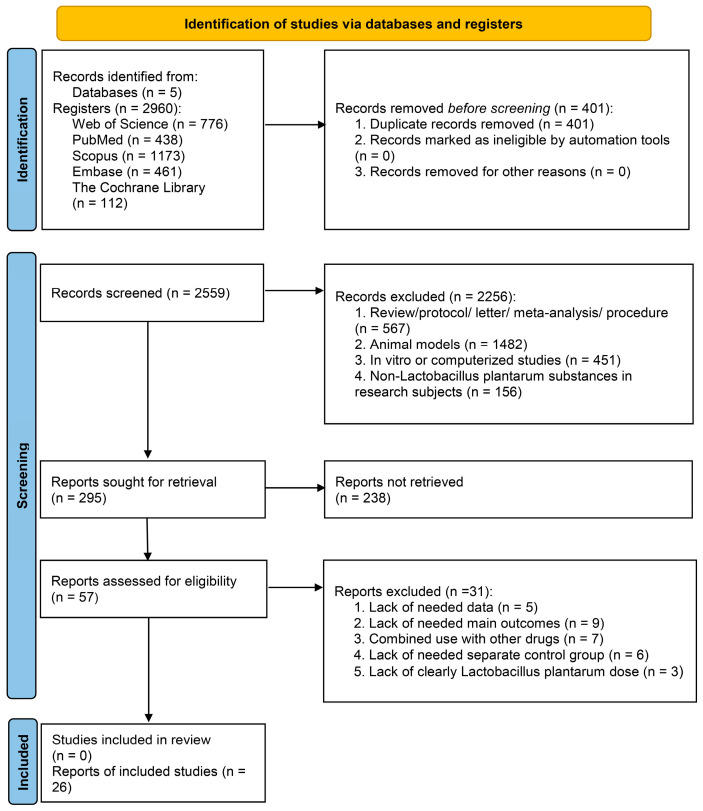
The flowchart of paper identification and the inclusion process.

Following a systematic literature search, 2960 records were initially identified. After importing the results into EndNote, 401 duplicates were removed through title and abstract screening, resulting in 57 articles that underwent full-text assessment. According to the inclusion and exclusion criteria, 31 articles were excluded due to *L. plantarum* being used with other drugs (*n* = 7), lack of clear use of *L. plantarum* (*n* = 3), lack of needed data (*n* = 5), lack of needed main outcomes (*n* = 9), and lack of needed separate control group (*n* = 6). In total, 26 studies were included in quantitative and qualitative analysis (Figure 1).

### 3.2. Characteristics of Participants and Studies

The characteristics of the 26 studies included in the meta-analysis are summarized in Table 1. Our pooled analysis comprised a total of 2104 participants from these trials, with 1071 subjects in the intervention group and 1033 subjects in the placebo control group. These studies were published between 2010 and 2024 and were conducted in Korea (*n* = 5) [23,33,34,35,36], Estonia (*n* = 3) [21,24,37], Indonesia (*n* = 2) [26,27], UK (*n* = 2) [26,38], Iran (*n* = 2) [39,40], Japan (*n* = 2) [41,42], Australia [43], Germany [22], Greece [44], Ukraine [45], Italy [46], USA [47], Brazil [48], Russia [49], Spain [50], and India [51]. The intervention time ranged from 3 weeks to 6 months, and the doses of *L. plantarum* ranged from 10^8.17^ to 7 × 10^10^ cfu/day. One of these studies focused on men only [48], three on women only [27,43,44], and the remaining 22 included both men and women. All 26 studies used the principle of random allocation, 23 were double-blind, and the remaining three did not report blinding [38,45,48]. Two were crossover studies [37,47], and the rest were parallel studies. Twenty-six studies included 30 intervention groups, of which 14 used probiotic dairy products [21,24,26,27,37,40,41,42,48,49], 14 used probiotic capsules [22,23,26,33,34,35,38,39,43,44,45,47,50,51], one used a probiotic beverage [46], and one used a probiotic powder [36]. Ten intervention groups used a probiotic supplement containing multiple strains of *L. plantarum*, while the remaining 20 intervention groups used a single supplement of *L. plantarum*.

### 3.3. Quality Assessment

Quality assessment of all studies included are provided in Figure S1. All 26 randomized controlled trials (RCTs) adequately described their methodologies for random sequence generation, and the majority reported details on allocation concealment. Furthermore, blinding of participants and outcome assessors was implemented in most studies. The risk of bias was generally low across domains, including incomplete outcome data, selective reporting, and other potential biases. However, a subset of trials exhibited unclear methodological descriptions in certain bias domains. Importantly, no studies were rated as having a high overall risk of bias.

### 3.4. Pooled Effect Size of L. plantarum Supplementation on TC

**Figure 2:**
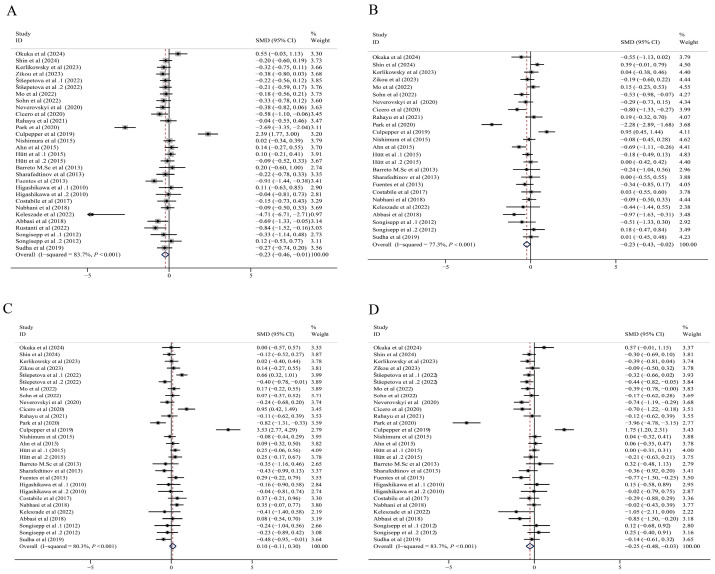
Forest plot of the effect of *L. plantarum* on blood lipids: (**A**) TC, (**B**) TG, (**C**) HDL-C, (**D**) LDL-C. Abbreviations: HDL-C, high-density lipoprotein cholesterol; LDL-C, low-density lipoprotein cholesterol; *Lactobacillus plantarum*, *L. plantarum*; SMD, standardized mean differences; TG, triglyceride; TC, total cholesterol [21,22,23,24,26,27,33,34,35,36,37,38,39,40,41,42,43,44,45,46,47,48,49,50,51].

In 26 studies, 2104 subjects reported TC levels as the main result. Figure 2A shows the effect of *L. plantarum* on TC. The combined results of the random effects model showed that *L. plantarum* supplementation had a positive effect on TC, reducing the TC values (SMD: −0.23 mg/dL; 95%CI: −0.46, −0.01; *p* = 0.042). There was significant heterogeneity among the studies (I^2^ = 83.7%; *p* < 0.001). Sensitivity analysis showed that removing either study did not change the direction of the effect (Figure S2A).

### 3.5. Pooled Effect Size of L. plantarum Supplementation on TG

Twenty-three studies evaluated 1763 participants and reported the TG. Figure 2B shows that *L. plantarum* supplementation had a positive benefit on TG. The combined results of the random effects model showed that *L. plantarum* supplementation significantly reduced the TG (SMD: −0.23 mg/dL; 95%CI: −0.43, −0.02; *p* = 0.030), with significant heterogeneity among the studies (I^2^ = 77.3%; *p* < 0.001). When any single study was removed, sensitivity analyses consistently preserved the directionality of the effect (Figure S2B).

### 3.6. Pooled Effect Size of L. plantarum Supplementation on HDL-C

A total of 2068 participants were evaluated in 25 studies, where HDL-C levels were reported as the main result. The results demonstrated that *L. plantarum* intake led to no evident variation in HDL-C levels (SMD: 0.10 mg/dL; 95% CI: −0.11, 0.03, *p* = 0.342: Figure 2C), with significant heterogeneity across studies (I^2^ = 80.3%, *p* < 0.001: Figure 2C). The direction of the effect remained unchanged in sensitivity analyses after sequentially excluding individual studies, as demonstrated in Figure S2C.

### 3.7. Pooled Effect Size of L. plantarum Supplementation on LDL-C

Figure 2D shows the results of the LDL-C assessment in 2068 participants from a total of 25 studies. The combined results indicated that *L. plantarum* supplementation caused a significant decrease in LDL-C levels (SMD: −0.25 mg/dL, 95% CI: −0.48, −0.03, *p* = 0.029). Significant heterogeneity was detected across these studies (I^2^ = 83.7%, *p* < 0.001). The robustness of the effect direction was confirmed by sensitivity analyses, wherein the exclusion of individual studies failed to alter the observed trends (Figure S2D).

### 3.8. Subgroup Analysis

Subgroup analyses were performed to identify potential sources of heterogeneity. We performed subgroup analysis of supplement duration and supplement composition (Table 2), and the results showed that, for a duration of less than 8 weeks, *L. plantarum* supplementation did not significantly reduce the serum TC, TG, and LDL-C levels, while for a duration of more than 8 weeks, there was a significant reduction in the serum TC (SMD: −0.386, 95%CI: 0.686, 0.058, *p* = 0.012), TG (SMD: 0.334, 95% CI: 0.647, 0.021, *p* = 0.036), and LDL—C (SMD: 0.466, 95% CI: −0.834, −0.098, *p* = 0.013). We also conducted subgroup analysis of *L. plantarum* supplement ingredients, and the results showed that the *L. plantarum* single-strain supplement showed a reduced serum TC (SMD: −0.406, 95% CI: −0.678, −0.135, *p* = 0.003), TG(SMD: 0.270, 95% CI: 0.527 0.013, *p* = 0.040), and LDL—C (SMD: 0.406, 95% CI: −0.700, −0.111, *p* = 0.007). However, the meta-analysis did not show that *L. plantarum* multi-strain supplements could reduce the serum TC, TG and LDL-C levels. Heterogeneity decreased in some of the subgroups, but remained significant.

### 3.9. Meta-Regression Analysis

A random-effects meta-regression was conducted to examine the influence of publication year, intervention group sample size, geographic region (categorical), mean baseline TC level, mean baseline age, and mean baseline BMI as covariates on the mean difference in lipid outcomes. The analysis revealed no statistically significant associations, and no clear linear relationships were detected between these variables and changes in lipid levels (Table S3).

### 3.10. Dose–Response Meta-Analysis

Figure S3 illustrates the dose–response relationship between *L. plantarum* supplementation and changes in TC, TG, HDL-C, and LDL-C. Since the administered doses ranged from 10^8^ to 10^10^ CFU/day, a natural logarithmic transformation was applied to normalize the dose scale across studies. No significant linear or nonlinear associations were observed between the log-transformed daily dose of *L. plantarum* and alterations in any of the lipid parameters (Table S4).

### 3.11. Publication Bias Assessment

**Figure 3:**
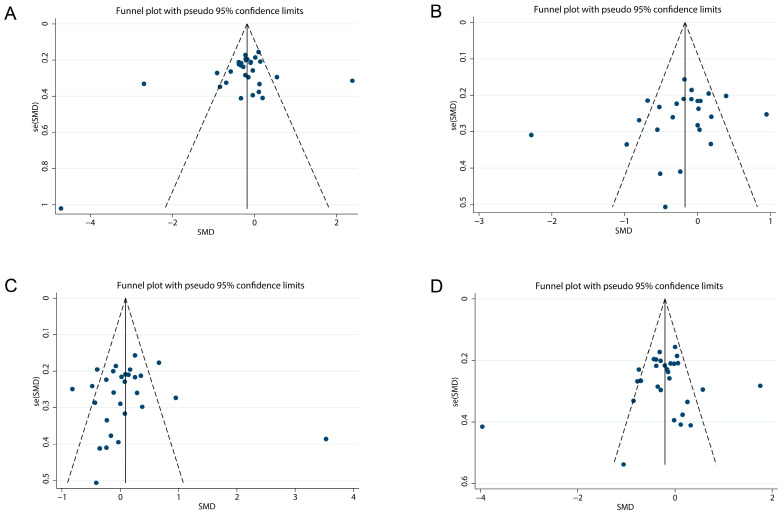
Funnel plot of the effect of *L. plantarum* on blood lipids: (**A**) TC, (**B**) TG, (**C**) HDL-C, (**D**) LDL-C. Abbreviations: HDL-C, high-density lipoprotein cholesterol; LDL-C, low-density lipoprotein cholesterol; *Lactobacillus plantarum*, *L. plantarum*; se (SMD), standard error (Standard Mean Difference); TG, triglyceride; TC, total cholesterol.

Potential publication bias was assessed through funnel plot symmetry and statistical evaluation using Egger’s and Begg’s tests. Visual inspection of the funnel plots revealed no asymmetry (Figure 3), indicating an absence of publication bias. Statistical analyses further supported these findings, with non-significant *p*-values for all lipid parameters (TC: Egger’s *p* = 0.224, Begg’s *p* = 0.284; TG: Egger’s *p* = 0.117, Begg’s *p* = 0.118; HDL-C: Egger’s *p* = 0.850, Begg’s *p* = 0.399; LDL-C: Egger’s *p* = 0.427, Begg’s *p* = 0.807).

### 3.12. Information on Mechanisms and Targets

**Figure 4:**
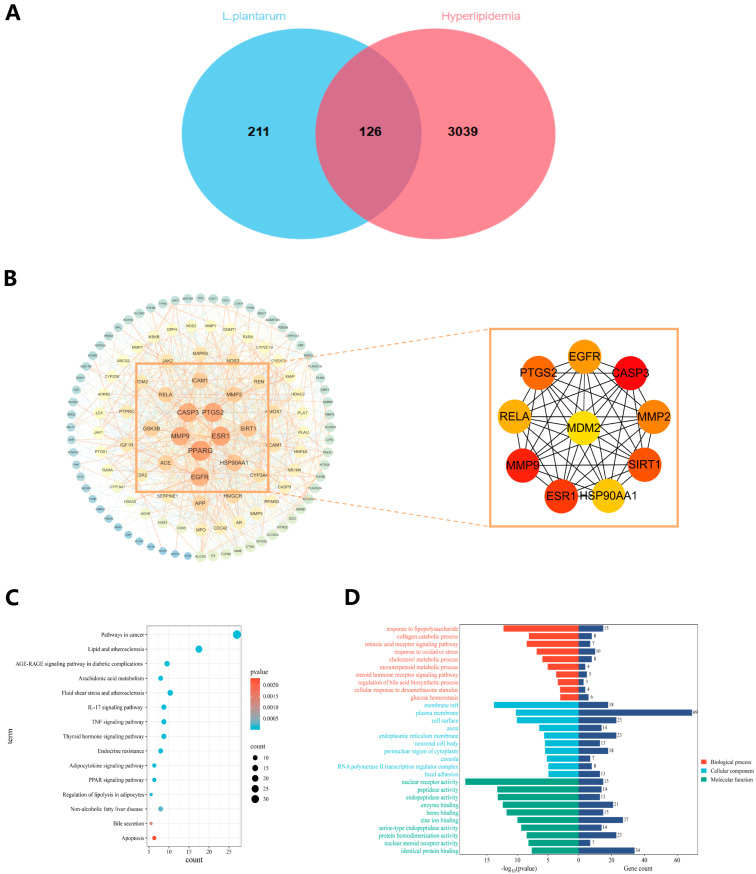
The potential targets and mechanisms of action of *L. plantarum* were predicted by network pharmacology. (**A**) Overlapping targets of *L. plantarum* targets and genes associated with hyperlipidemia. (**B**) The PPI network of overlapping targets. The red nodes represent the core targets. (**C**) Bar chart of KEGG enrichment analysis. (**D**) GO enrichment analysis bar chart. Abbreviations: *Lactobacillus plantarum*, *L. plantarum*; PPI, protein–protein interaction; BP, biological process; CC, cellular component; GO, Gene Ontology; KEGG, Kyoto Encyclopedia of Genes and Genomes; MF, molecular function.

To elucidate the molecular mechanisms underlying the lipid-lowering effects of *L. plantarum* metabolites, we conducted protein–protein interaction (PPI) network analysis and functional enrichment analysis on both metabolite targets and hyperlipidemia-related targets. Metabolite targets of *L. plantarum* were retrieved from the Swiss Target Prediction and TargetNet databases, yielding 337 unique targets after deduplication. Disease targets associated with hyperlipidemia were collected from OMIM and GeneCards using “hyperlipidemia” as the keyword, resulting in 3165 non-redundant targets. Intersection analysis identified 126 overlapping targets (Figure 4A), which were proposed as prospective targets for the hypolipidemic activity of *L. plantarum*. The PPI network of overlapping targets was constructed using the STRING database. As shown in Figure 4B, the network comprised 126 nodes and 1130 edges. To refine the core targets, the Maximal Clique Centrality (MCC) algorithm was applied, revealing top-ranked targets such as PPARG, MMP9, and CASP3. Kyoto Encyclopedia of Genes and Genomes (KEGG) enrichment analysis highlighted 15 potential pathways mediating the lipid-lowering effects of *L. plantarum* (Figure 4C), including IL-17 signaling, TNF signaling, and bile secretion pathways. Gene Ontology (GO) analysis further delineated the biological roles of these targets across three categories. Notably, key enriched GO terms included “response to lipopolysaccharide,” “membrane raft,” and “nuclear receptor activity” (Figure 4D). These findings collectively suggest that *L. plantarum* metabolites may ameliorate hyperlipidemia by regulating the oxidative stress inflammatory pathway, bile secretion pathway, and lipid metabolism-related targets.

## 4. Discussion

Previous studies have confirmed the blood pressure-modulating potential of *L. plantarum* [28]. This meta-analysis further reveals its significant role in improving lipid metabolism. To our knowledge, this systematic review is the first to synthesize the evidence on the effects of *L. plantarum*-containing probiotic supplements on blood lipid profiles. Although numerous clinical trials have investigated the impact of *L. plantarum* on lipid concentrations, the results remain inconsistent. Our analysis incorporated data from 26 eligible randomized controlled trials (RCTs). The meta-analysis results demonstrated that *L. plantarum* supplementation exerts significant beneficial effects on total cholesterol (TC), triglycerides (TG), and low-density lipoprotein cholesterol (LDL-C). Subgroup analyses further indicated that intervention durations exceeding 8 weeks and single-strain *L. plantarum* formulations exhibit more pronounced effects on TC, TG, and LDL-C reduction. Additionally, we pioneered the application of network pharmacology to explore the mechanistic basis of *L. plantarum*’s lipid-lowering effects. The results suggest that *L. plantarum* likely ameliorates dyslipidemia through pathways involving bile acid metabolism, antioxidant activity, and anti-inflammatory modulation. These insights advance our understanding of probiotic-mediated lipid regulation and underscore the therapeutic potential of targeting gut microbiota–host interactions in metabolic disorders.

Hyperlipidemia is widely recognized as an independent risk factor for CVDs and metabolic disorders [52,53]. Notably, dyslipidemia is not exclusive to individuals with overweight or obesity. Even those with normal or low body weight may exhibit abnormal lipid profiles, often overlooking the importance of lipid profile monitoring, thereby increasing their susceptibility to severe cardiovascular complications [54]. In recent years, the role of probiotics in modulating host metabolism has garnered significant attention [55]. Probiotics can exert various beneficial functions in the body, and more and more evidence indicates that probiotic supplementation exerts beneficial effects on atherosclerosis, cardiovascular diseases, and inflammatory conditions [12]. *L. plantarum* is a type of probiotic commonly used in nutritional supplements and is widely employed in the food and dairy industries. It has demonstrated potential in ameliorating metabolic disturbances and cardiovascular risks [56,57]. Preclinical studies in animal models reveal that *L. plantarum* supplementation regulates lipid levels in obese or hypercholesterolemic mice [18,19]. Li et al. proposed that this effect may stem from the modulation of gut microbiota and lipid metabolism-associated metabolites [58]. An earlier meta-analysis investigating probiotic effects on lipid profiles indicated that pooled results from three *L. plantarum*-specific studies significantly reduced total cholesterol (TC) and low-density lipoprotein cholesterol (LDL-C) [59], aligning with our findings. Our analysis further extends these benefits to triglyceride (TG) reduction. However, a recent meta-analysis focusing on *L. plantarum* supplementation in prediabetic and type 2 diabetic populations reported no significant lipid-lowering effects [60]. This discrepancy may reflect the limited generalizability of the three included RCTs in diabetic cohorts. While previous meta-analyses on probiotics and lipid metabolism have yielded inconsistent conclusions, our study—incorporating a larger body of evidence—demonstrates robust benefits of *L. plantarum* supplementation on TC, TG, and LDL-C reduction. These findings may provide a theoretical basis for developing targeted probiotic interventions for hyperlipidemia prevention and management. Subgroup analyses revealed that intervention durations of 8 weeks or longer significantly enhanced the TC, TG, and LDL-C reduction, whereas shorter regimens showed negligible effects, underscoring the necessity of sustained *L. plantarum* supplementation for therapeutic efficacy. Stratified analysis by probiotic formulation type demonstrated that single-strain *L. plantarum* interventions conferred marked lipid-lowering benefits, while multi-strain formulations lacked significant effects. This observation parallels findings from a prior meta-analysis on *L. plantarum* and blood pressure regulation, where single-strain preparations outperformed multi-strain combinations [28] Collectively, these results highlight the need to prioritize specific well-characterized *L. plantarum* strains for cost-effective and potent lipid management strategies.

While significant reductions were observed in TC, TG, and LDL-C, no notable effect was seen on HDL-C—a discrepancy that may be attributed to several complicating factors. First, HDL-C metabolism is highly complex and exhibits considerable interindividual variation. Genetic background, baseline HDL-C concentrations, as well as differences in HDL particle composition and functionality may all help explain the lack of detectable change [61,62]. Second, the impact of *L. plantarum* on HDL-C may be modulated by lifestyle factors such as diet quality and physical activity, which have been shown in previous studies to significantly influence HDL-C levels [63,64]. Third, the formulation of the probiotic supplement may also play a role. Evidence suggests that dairy-based carriers can enhance probiotic survival through gastrointestinal transit and improve efficacy compared to capsule-based supplements [65]. Furthermore, as highlighted in a review, microbial interventions tend to exhibit lipid-specific effects, and not all lipid parameters respond equally to probiotic treatment [66].

The results of the subgroup analysis still showed high heterogeneity, and the meta-regression analysis discussed in depth the year of publication, sample size, region, baseline lipid level, age, and baseline BMI as covariates. None of the covariates tested were statistically significant in explaining the heterogeneity observed for the four lipid outcomes in the meta-analysis. This may be attributed to other more complex factors, including the following: Strain-specific effects, particularly differences in the efficacy among subspecies or strains of *L. plantarum*, may not have been fully accounted for in our analysis. Additionally, the background diet of the study populations—a factor known to significantly modulate probiotic impacts—was often insufficiently detailed in the original studies and did not permit meaningful inclusion in the meta-analysis. Our RCS analysis indicated that the lipid-lowering effects of *L. plantarum* were observed across a broad dosage range (10^8^–10^10^ CFU/day), with no clear “more is better” relationship detected. This finding holds clinical relevance, as it suggests that even doses at the lower end of this spectrum may be sufficient to confer benefits—potentially enhancing cost-effectiveness and improving patient compliance.

Currently, there is very little research on the mechanism by which *L. plantarum* lowers blood lipids. Several research studies have demonstrated that *L. plantarum* exerts its probiotic functions and assumes an anti-obesity role through the regulation of the gut microbiota [67,68]. Cai et al. found that natto yogurt fermented by *L. plantarum* could inhibit fatty acid synthesis and enhance fatty acid catabolism by regulating the expression of PPARα, PPARγ, CD36, and FAS in the liver, thereby exerting the effect of lowering blood lipids [69]. Another study found that *L. plantarum* could activate the TLR4/NF-κB signaling pathway, reduce intestinal inflammation, enhance the intestinal barrier function, and decrease lipid accumulation [19]. We further explored the mechanism of the blood lipid lowering of *L. plantarum* using network pharmacology. We conducted PPI, KEGG, and GO analyses on the metabolites of *L. plantarum* in the human body and the overlapping targets of hyperlipidemia. Among the core genes screened by the PPI results, we discovered the core target PPARG. PPARG is related to adipocyte differentiation, lipid storage, and insulin sensitivity [70,71]. Some studies have shown that it is associated with the changes in blood lipids caused by atherosclerosis [72,73]. MMP9, one of the core genes, is also closely related to changes in blood lipids. Studies have shown that oxidized low-density lipoprotein (oxLDL) upregulates the transcription of the MMP9 gene by activating the LOX-1 receptor and the NF-κB pathway [74]. High-density lipoprotein (HDL) inhibits the activity of MMP9 through apolipoprotein A-I (ApoA-I). In patients with low HDL levels, the increased activity of MMP9 increases the risk of cardiovascular events [75]. This suggests that *L. plantarum* may exert the effect of lowering blood lipids by regulating key targets such as PPARG and MMP9. The KEGG results show that significant pathways included oxidative stress and inflammatory pathways such as IL-17 and TNF. As is well known, oxidative stress and inflammation interact with and influence each other in the elevation of blood lipids and play a key role in pathological processes such as cardiovascular diseases [74,75]. This suggests that *L. plantarum* can exert anti-inflammatory and antioxidant effects and thus regulate the changes in blood lipids. We also found that pathways such as the adipocytokine signaling pathway, regulation of lipolysis in adipocytes, and bile secretion play key roles in this process. It is found that *L. plantarum* may regulate lipid changes by promoting the metabolism and decomposition of fatty acids and fats in adipocytes and through the pathway of bile secretion. The exploration of the mechanism by network pharmacology may provide some ideas for future research on the mechanism of blood lipid lowering by *L. plantarum*, suggesting that the mechanism of the blood lipid-lowering effect of *L. plantarum* may be influenced by multiple aspects in an intersecting manner.

Certainly, our meta-analysis has several inevitable limitations. Firstly, despite our attempts to elucidate the sources of heterogeneity, a significant portion remained unexplained—a common limitation in nutritional meta-analyses. This underscores the necessity for future primary studies to report more consistently on these potential effect modifiers. Secondly, the included studies were in English, which may lead to the omission of relevant studies from non-English sources. Finally, the pooling of data from studies involving different strains and formulations of Lactobacillus plantarum represents a significant source of clinical and methodological heterogeneity. These subspecies and strains often exhibit strain-specific properties and efficacy due to underlying genetic variability. Moreover, the use of various delivery substrates—such as yogurt or other fermented foods—further complicates the interpretation, as the carrier vehicles themselves may influence lipid metabolism or modulate the gut microbiome.

## 5. Conclusions

This study consolidates evidence from 26 RCTs, demonstrating that *L. plantarum* supplementation effectively reduces TC, TG, and LDL-C levels, particularly when administered as a single strain and over durations exceeding 8 weeks. The integration of network pharmacology revealed potential mechanisms involving anti-inflammatory pathways (e.g., IL-17/TNF signaling), nuclear receptor regulation (e.g., PPARG), and bile acid metabolism. These insights underscore the therapeutic potential of *L. plantarum* in managing dyslipidemia and mitigating cardiovascular risks. However, the heterogeneity across studies, the variability in strains, and the formulation differences highlight the need for standardized protocols in future research. Further investigations should prioritize strain-specific effects, optimal dosing, and mechanistic validation through preclinical models. The results of this study provide a solid scientific basis for the development of functional foods and dietary supplements based on Lactobacillus plantarum. It can be used as an auxiliary method for managing and treating hyperlipidemia.

## Figures and Tables

**Table 1:** Characteristics of included trials [21,22,23,24,26,27,33,34,35,36,37,38,39,40,41,42,43,44,45,46,47,48,49,50,51].

Author	Year	References	Design of Studies	Country	No. of Subjects in Case Group	No. of Controls	Gender	Age (Mean)	Follow-up Duration	Clinical Condition	Dosage (Daily)	Outcomes
Okuka	2024	[43]	Double-blind placebo-controlled supplementary intervention study	Australia	25	23	F	39.33	12 weeks	Obese women	One probiotic capsule containing 7 × 10^10^ cfu of *Lactobacillus plantarum* (*L. plantarum*) 299v	TC, TG, HDL-C, LDL-C
Shin	2024	[23]	Double-blind randomized clinical trial	Korea	50	50	M F	48.90	12 weeks	Healthy adults	One probiotic capsule containing 2 × 10^10^ cfu of *L. plantarum* SKO-001	TC, TG, HDL-C, LDL-C
Kerlikowsky	2023	[22]	Double-blind randomized placebo-controlled nutritional intervention trial	Germany	43	43	M F	63.6	12 weeks	Hypercholesterolemia	One probiotic capsule containing 1.2 × 10^9^ cfu of *L. plantarum* CECT7527 (KABP011), *L. plantarum* CECT7528 (KABP012), and *L. plantarum* CECT7529 (KABP013)	TC, TG, HDL-C, LDL-C
Zikou	2023	[44]	Single-center double-blind placebo-controlled randomized clinical trial	Greece	46	45	M F	64.54	6 months	Type 2 diabetes mellitus	One probiotic capsule containing 1.75 × 10^9^ cfu of Lactobacillus acidophilus, 0.5 × 10^9^ cfu of *L. plantarum* 1.75 × 10^9^ cfu of Bifidobacterium lactis, and 1.5 × 10^9^ cfu of Saccharomyces boulardii	TC, TG, HDL-C, LDL-C
Štšepetova 1 (JOG 4BC)	2023	[24]	Two parallel two-armed double-blind placebo-controlled (DBPC) human intervention trials	Estonia	73	63	M F	42.14	8 weeks	Healthy adults	150 g of probiotic yoghurt containing 5.9 × 10^9^ cfu of *L. plantarum* Inducia, 6.6 × 10^10^ cfu of Lactobacillus delbrueckii subsp, and 6.75 × 10^9^ cfu of Streptococcus thermophilus	TC, TG, HDL-C, LDL-C
Štšepetova 2 (JOG5)	2023	[24]	Two parallel two-armed double-blind placebo-controlled (DBPC) human intervention trials	Estonia	53	52	M F	46.74	8 weeks	Healthy adults	150 g of probiotic yoghurt containing 2.0 × 10^9^ cfu of *L. plantarum* Inducia, 6.6 × 10^10^ cfu of Lactobacillus delbrueckii subsp, and 6.75 × 10^9^ cfu of Streptococcus thermophilus	TC, TG, HDL-C, LDL-C
Mo	2022	[33]	Randomized double-blind placebo-controlled study	Korea	30	29	M F	35.7	12 weeks	Healthy obese and overweight adults	One probiotic capsule containing 5 × 10^9^ cfu of *L. plantarum* KY1032	TC, TG, HDL-C, LDL-C
Sohn	2022	[34]	Randomized double-blind placebo-controlled clinical trial	Korea	41	36	M F	47.8	12 weeks	Healthy adults	Two probiotic capsules containing 4 × 10^9^ cfu of *L. plantarum* K50 (LPK)	TC, TG, HDL-C, LDL-C
Neverovskyi	2021	[45]	The present open comparative randomized parallel investigation	Ukraine	41	40	M F	57.7	12 weeks	Dyslipidemia	One probiotic capsule containing 2 × 10^9^ cfu of *L. plantarum*	TC, TG, HDL-C, LDL-C
Cicero	2020	[46]	Double-blind randomized placebo-controlled parallel-group clinical trial	Italy	30	30	M F	72	2 months	Elderly patients with a diagnosis of MetS	One liquid vial containing 2 × 10^9^ cfu of *L. plantarum* PBS067—DSM 24,937, 2 × 10^9^ cfu of Lactobacillus acidophilus PBS066—DSM 24,936, and 2 × 10^9^ cfu of Lactobacillus reuteri PBS072—DSM 25,175	TC, TG, HDL-C, LDL-C
Rahayu	2021	[26]	Randomized double-blind placebo-controlled study	Indonesia	30	30	M F	44.07	3months	BMI ≥ 25	1 g of skimmed milk powder containing 2 × 10^9^ cfu of *L. plantarum* Dad-13	TC, TG, HDL-C, LDL-C
Park	2020	[35]	Randomized double-blind placebo-controlled parallel trial	Korea	35	35	M F	48.3	12 weeks	TG < 200 mg/dL	Two 400 mg probiotic capsules containing 4.0 × 10^9^ cfu of *L. plantarum* (LPQ180)	TC, TG, HDL-C, LDL-C
Culpepper	2019	[47]	Randomized double-blind placebo controlled crossover study	USA	35	35	M F	54.3	18 weeks	Healthy adults	One probiotic capsule containing 5 × 10^9^ cfu of *L. plantarum* HA-119, 2.5 × 10^9^ cfu of B. subtilis R0179, and 5 × 10^9^ cfu of B. lactis B94	TC, TG, HDL-C, LDL-C
Nishimura 1 (Placebo 1)	2015	[41]	Randomized double-blind placebo-controlled study	Japan	57	55	M F	49.58	8 weeks	NK cell activity below 50%	90 g of yogurt containing *L. plantarum* HOKKAIDO ≥ 5.0 × 10^9^ cfu	TC, TG, HDL-C, LDL-C
Nishimura 2 (Placebo 2)	2015	[41]	Randomized double-blind placebo-controlled study	Japan	57	59	M F	49.58	8 weeks	NK cell activity below 50%	90 g of yogurt containing *L. plantarum* HOKKAIDO ≥ 5.0 × 10^9^ cfu	TC, TG, HDL-C, LDL-C
Ahn	2015	[36]	Randomized double-blind placebo-controlled study	South Korea	46	46	M F	54.1	12 weeks	Nondiabetic and hypertriglyceridemic subjects	2 g of powder containing 5 × 10^9^ cfu of L. curvatus HY7601 and 5 × 10^9^ cfu of *L. plantarum* KY1032	TC, TG, HDL-C, LDL-C
Hütt 1 (Chess trial)	2015	[21]	Two double-blind randomized placebo-controlled exploratory trials	Estonia	82	82	M F	37.7	3 weeks	Healthy adults	50 g of probiotic cheese containing 10^10^ cfu of *L. plantarum* TENSIA	TC, TG, HDL-C, LDL-C
Hütt 2 (Yoghurt trial)	2015	[21]	Two double-blind randomized placebo-controlled exploratory trials	Estonia	43	43	M F	34.2	3 weeks	Healthy adults	150 g of probiotic yoghurt containing 6 × 10^9^ cfu of *L. plantarum* TENSIA	TC, TG, HDL-C, LDL-C
Barreto	2014	[48]	Randomized placebo-controlled study	Brazil	12	12	M	62	3 months	Postmenopausal women with MetS	80 mL of fermented milk [FM])containing 10^9^ cfu of *L. plantarum* (LP115)	HDL-C, LDL-C
Sharafedtinov	2013	[49]	Randomized double-blind placebo-controlled parallel pilot study	Russia	25	15	M F	52.0	3 weeks	Metabolic syndrome characterized by obesity accompanied by arterial hypertonia	50 g of probiotic product (semi-hard cheese) containing 7.5 × 10^12^ cfu of *L. plantarum* TENSIA	TC, TG, HDL-C, LDL-C
Fuentes	2012	[50]	Single-center prospective randomized double-blind placebo-controlled parallel-group trial	Spain	30	30	M F	NA	12 weeks	Healthy adults	One probiotic capsule containing 1.2 × 10^9^ cfu of *L. plantarum* (CECT 7527, CECT 7528 and CECT 7529)	TC, HDL-C, LDL-C
Higashikawa 1(Group A)	2010	[42]	Double-blind randomized design with three parallel groups	Japan	24	20	M F	37.3	6 weeks	Healthy adults	100 g of probiotic yoghurt containing 1.9 × 10^10^ cfu of *L. plantarum* SN35N and 10^9^ cfu of *L. plantarum* SN13T	TC, HDL-C, LDL-C
Higashikawa 2(Group B)	2010	[42]	Double-blind randomized design with three parallel groups	Japan	18	20	M F	35.1	6 weeks	Healthy adults	100 g of probiotic yoghurt containing 1.96× 10^10^ cfu of *L. plantarum* SN13T and 4 × 10^8^ cfu of *L. plantarum* SN35N	TC, HDL-C, LDL-C
Costabile	2017	[38]	Single-center prospective randomized placebo-controlled parallel-group design	United Kingdom	23	23	M F	52.3	12 weeks	Normal to mildly hypercholesterolemic adults	Two probiotic capsules containing 4 × 10^9^ cfu of *L. plantarum* ECGC 13110402	TC, HDL-C, LDL-C
Nabhani	2018	[39]	Double-blind placebo-controlled randomized clinical trial	Iran	45	45	F	29.4	6 weeks	Pregnant women	One symbiotic capsule containing 2.5 × 10^10^ cfu of L. acidophilus, 7.5 × 10^9^ cfu of *L. plantarum*, 3.5 × 10^9^ cfu of L. fermentum, and 10^10^ cfu of L. gasseri	TC, TG, HDL-C, LDL-C
Keleszade	2022	[25]	Parallel double-blind placebo-controlled randomized pilot study	United Kingdom	8	8	M F	NA	6 weeks	Hypercholesterolemic adults	One probiotic capsule containing 4 × 10^9^ cfu of *L. plantarum* ECGC 13,110,402	TC, TG, HDL-C, LDL-C
Abbasi	2018	[40]	Parallel design double-blind randomized clinical trial	Iran	20	20	M F	56.9	8 weeks	Type 2 diabetic patients with nephropathy	200 mL of probiotic soy milk containing 4 × 10^9^ cfu of *L. plantarum* A7	TC, TG, HDL-C, LDL-C
Rustanti	2022	[27]	Randomized double-blind controlled trial	Indonesia	10	8	F	44.11	12 weeks	T2D patients	1 g skim milk powder containing 10^10^ cfu of *L. plantarum* Dad-13	TC
Songisepp (Study 1)	2012	[37]	Double-blind placebo-controlled (DBPC) crossover study	Estonia	12	12	M F	29.1	8 weeks	Healthy adults	50 g of the test cheese containing 10^10.4^ cfu of *L. plantarum* Tensia	TC, TG, HDL-C, LDL-C
Songisepp (Study 2)	2012	[37]	Double-blind placebo-controlled (DBPC) crossover study	Estonia	18	18	M F	69.8	8 weeks	Healthy elderly participants	50 g of the test cheese containing 10^8.17^ cfu of *L. plantarum* Tensia	TC, TG, HDL-C, LDL-C
Sudha	2019	[51]	Double-blind randomized parallel group placebo-controlled clinical trial	India	35	36	M F	43.5	12 weeks	Body mass index (BMI) 25–32 kg/m^2^	Two multi-strain probiotic capsules containing Lactobacillus salivarius UBLS-22, Lactobacillus casei UBLC-42, *L. plantarum*, UBLP-40, Lactobacillus acidophilus UBLA-34, Bifidobacterium breve UBBr-01, and Bacillus coagulans Unique IS 5 × 10^9^ cfu each	TC, TG, HDL-C, LDL-C

**Table 2:** Subgroup analysis.

Subgroup						
			SMD (95% CI)	Test for Overall Effect	Test for Heterogeneity	I^2^ (%)
Duration of study, weeks	≤8 weeks					
		TC	−0.073 (−0.404,0.257)	0.663	<0.001	84.60
		TG	−0.080 (−0.324,0.165)	0.525	0.003	62.50
		HDL-C	0.212 (−0.138,0.562)	0.235	<0.001	86.60
		LDL-C	−0.050 (−0.314,0.214)	0.709	<0.001	76.60
	>8 weeks					
		TC	−0.386 (−0.686,−0.058)	0.012	<0.001	81.70
		TG	−0.334 (−0.647,−0.021)	0.036	<0.001	82.70
		HDL-C	−0.012 (−0.217,0.193)	0.911	0.002	60.80
		LDL-C	−0.466 (−0.834,−0.098)	0.013	<0.001	87.20
Supplement	*L. plantarum*					
		TC	−0.406 (−0.678,−0.135)	0.003	<0.001	80.20
		TG	−0.270 (−0.527,−0.013)	0.040	<0.001	77.00
		HDL-C	−0.057 (−0.193,0.078)	0.407	0.164	24.10
		LDL-C	−0.406 (−0.700,−0.111)	0.007	<0.001	83.00
	Multi-strain probiotic					
		TC	0.086 (−0.310,0.482)	0.672	<0.001	87.90
		TG	−0.145 (−0.507,0.218)	0.434	<0.001	80.40
		HDL-C	0.457 (−0.025,0.938)	0.063	<0.001	91.50
		LDL-C	0.008 (−0.348,0.364)	0.965	<0.001	85.20

## Data Availability

No new data were created or analyzed in this study. Data sharing is not applicable.

## Supplementary Materials

The following supporting information can be downloaded at: https://www.mdpi.com/article/10.3390/foods14193300/s1, Figure S1 Summary of Risk of Bias for Included Studies. Figure S2. Results of sensitivity analysis in which the meta-analysis is re-estimated omitting each study in turn. Figure S3. Dose-response relationship between Lactobacillus plantarum dose and lipid change values. Table S1. Pathways enriched in GO analysis. Table S2. Pathways enriched in KEGG analysis. Table S3. Meta-regression effect size of blood lipids and moderator variables. Table S4. Dose–response analysis parameters. File S1. PRISMA_2020_checklist. Ref. [76] is cited in File S1.
