# Supplementary material for: Effect of Probiotics Containing Lactobacillus plantarum on Blood Lipids: Systematic Review, Meta-Analysis, and Network Pharmacological Analysis
Source: Foods. 2025 Sep 23;14(19):3300. doi: 10.3390/foods14193300 (PMC12523939; doi:10.3390/foods14193300)
Supplement: Supplementary file 1 [file foods-14-03300-s001.zip › Supplementary Material.pdf]

Search strategy

Pubmed

#1 (Lactobacillus plantarum [MeSH Terms]) OR (Lactobacillus plantarum [Title/Abstract]) OR (L. plantarum [Title/Abstract])

#2 (cholesterol[MeSH Terms]) OR (total cholesterol[MeSH Terms]) OR (Lipoproteins, HDL [MeSH Terms]) OR (Lipoproteins, LDL [MeSH Terms]) OR (triglycerides[MeSH Terms]) OR (triacylglycerol[MeSH Terms]) OR (blood lipids[MeSH Terms]) OR (Cholesterol, HDL [MeSH Terms]) OR (Cholesterol, LDL [MeSH Terms]) OR (Cholesterol, VLDL [MeSH Terms]) 543395

#3 (cholesterol[Title/Abstract]) OR (total cholesterol[Title/Abstract]) OR (high-density lipoprotein[Title/Abstract]) OR (low-density lipoprotein[Title/Abstract]) OR (triglycerides[Title/Abstract]) OR (triacylglycerol[Title/Abstract]) OR (blood lipids[Title/Abstract]) OR (high-density lipoprotein cholesterol[Title/Abstract]) OR (low-density lipoprotein cholesterol[Title/Abstract]) OR (Lipoproteins, HDL [Title/Abstract]) OR (Lipoproteins, LDL [Title/Abstract]) OR (Cholesterol, HDL[Title/Abstract]) OR (Cholesterol, LDL [Title/Abstract]) OR (Cholesterol, VLDL[Title/Abstract]) 370220

#1 AND (#2 OR #3)

Scopus

((TITLE-ABS-KEY (Lactobacillus plantarum) OR TITLE-ABS-KEY (L. plantarum) AND (TITLE-ABS-KEY (cholesterol) OR TITLE-ABS-KEY (total AND cholesterol) OR TITLE-ABS-KEY (lipoproteins, AND hdl) OR TITLE-ABS-KEY (lipoproteins, AND ldl) OR TITLE-ABS-KEY (triglycerides) OR TITLE-ABS-KEY (triacylglycerol) OR TITLE-ABS-KEY (blood AND lipids) OR TITLE-ABS-KEY (cholesterol, AND hdl) OR TITLE-ABS-KEY (cholesterol, AND ldl))

Web of science

TS= (Lactobacillus plantarum OR L. plantarum) AND TS=(cholesterol OR total cholesterol OR high-density lipoprotein OR low-density lipoprotein OR triglycerides OR triacylglycerol OR blood lipids OR high-density lipoproten cholesterol OR low-density lipoprotein cholesterol)

Embase

('Lactobacillus plantarum'/exp OR 'L. plantaricin'/exp OR L. plantaricin:ti,ab OR Lactobacillus plantarum:ab,ti) AND ('cholesterol'/exp OR 'cholesterol blood level'/exp OR 'high density lipoprotein'/exp OR 'low density lipoprotein'/exp OR 'triacylglycerol'/exp OR 'high density lipoprotein cholesterol'/exp OR 'low density lipoprotein cholesterol'/exp OR 'very low density lipoprotein cholesterol'/exp OR cholesterol:ab,ti OR 'total cholesterol':ab,ti OR 'lipoproteins, ldl':ab,ti OR 'triglycerides':ab,ti OR 'triacylglycerol':ab,ti OR 'blood lipids':ab,ti OR 'cholesterol, hdl':ab,ti OR 'cholesterol, ldl':ab,ti OR 'cholesterol, vldl':ab,ti)

Cocarane library

#1 (Lactobacillus plantarum):ti,ab,kw OR (L. plantarum):ti,ab,kw

#2 (cholesterol):ti,ab,kw OR (total cholesterol):ti,ab,kw OR (high-density lipoprotein):ti,ab,kw OR (low-density lipoprotein):ti,ab,kw OR (triglycerides):ti,ab,kw OR (triacylglycerol):ti,ab,kw OR

(blood lipids):ti,ab,kw OR (high-density lipoprotein cholesterol):ti,ab,kw OR (low-density lipoprotein cholesterol):ti,ab,kw OR (Lipoproteins, HDL):ti,ab,kw OR (Lipoproteins, LDL):ti,ab,kw OR (Cholesterol, HDL):ti,ab,kw OR (Cholesterol, LDL):ti,ab,kw OR (Cholesterol, VLDL):ti,ab,kw

#3: #1 AND #2

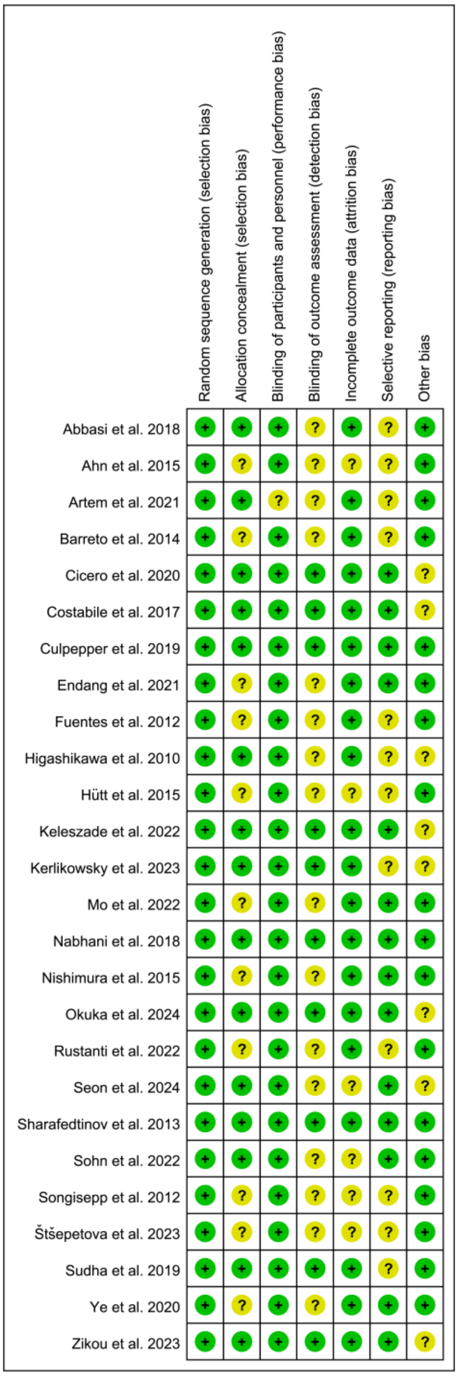

Figure S1 Summary of Risk of Bias for Included Studies.

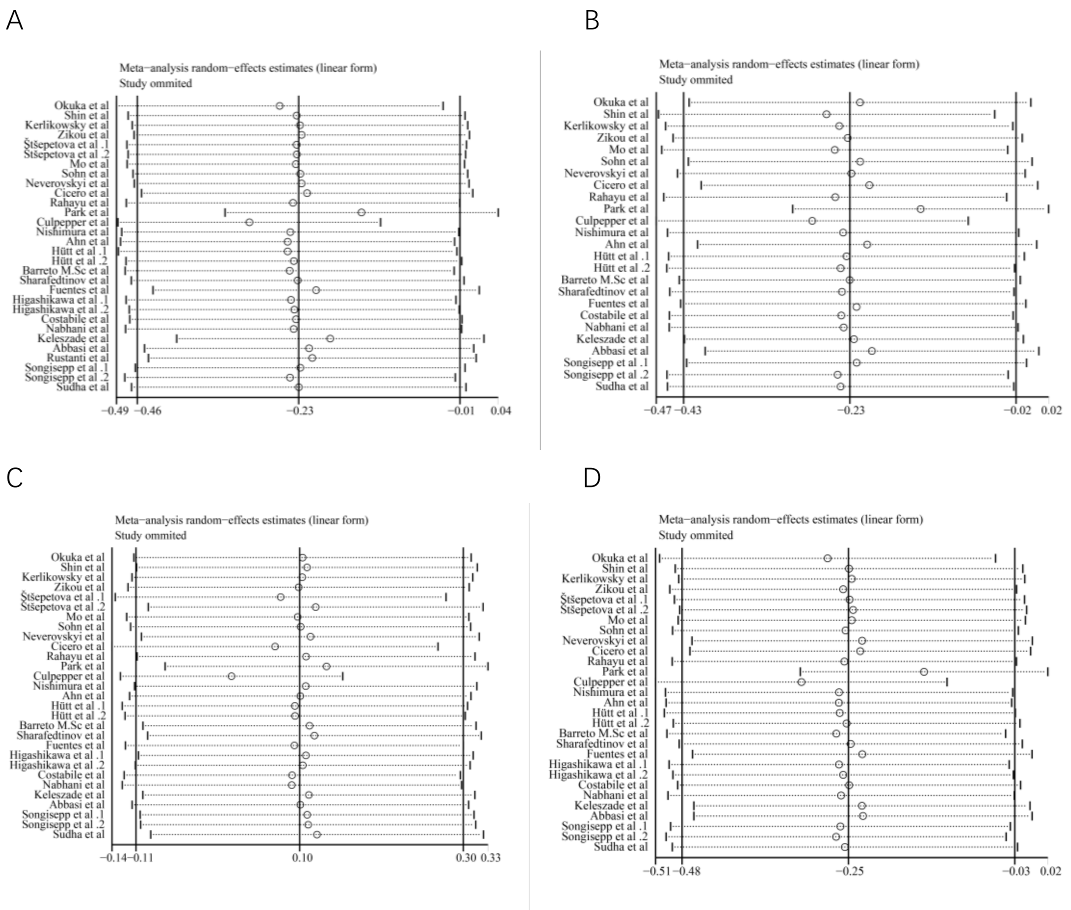

Figure S2. Results of sensitivity analysis in which the meta-analysis is re-estimated omitting each study in turn. (A) TC, (B) TG, (C) HDL-C, (D) LDL-C.

Abbreviations: HDL-C, high-density lipoprotein cholesterol; LDL-C, low-density lipoprotein cholesterol; TG, triglyceride; TC, total cholesterol.

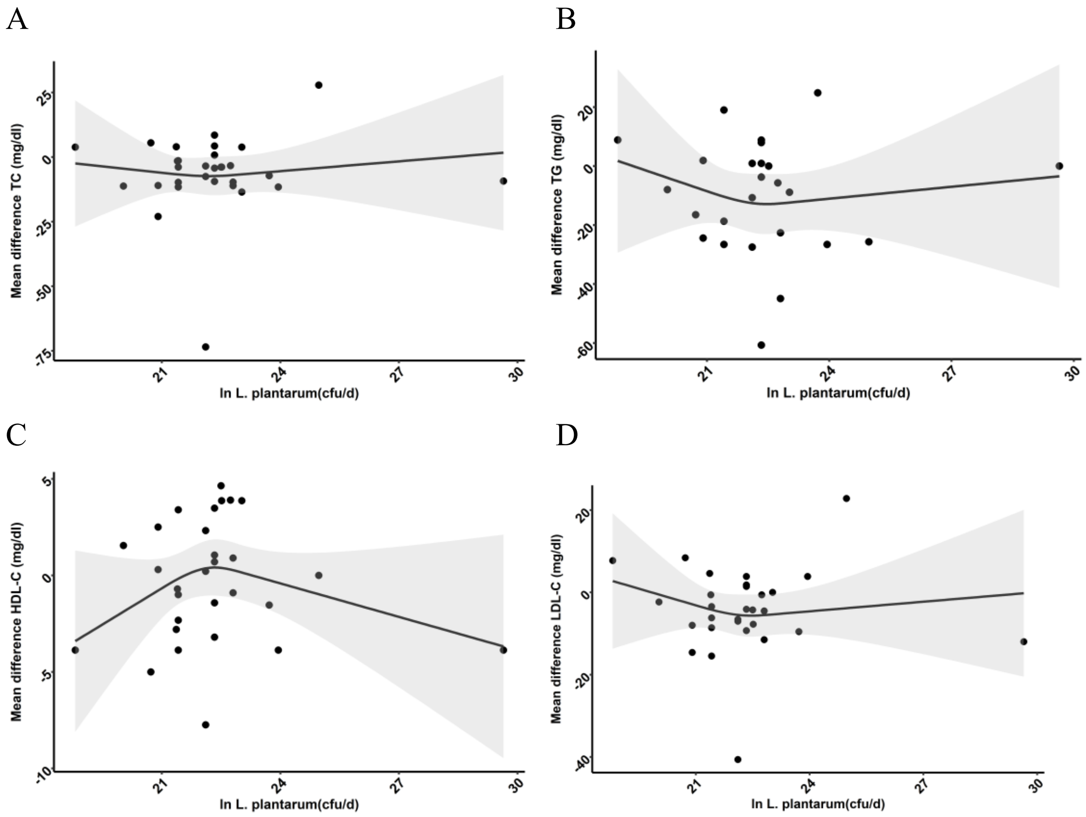

Figure S3. Dose-response relationship between Lactobacillus plantarum dose and lipid change values. (A) TC, (B) TG, (C) HDL-C, (D) LDL-C.

Abbreviations: HDL-C, high-density lipoprotein cholesterol; LDL-C, low-density lipoprotein cholesterol; TG, triglyceride; TC, total cholesterol.

Table S1. Pathways enriched in GO analysis

Category	Description	Gene ratio	PValue	Count
BP	response to lipopolysaccharide	11.90	5.35E-13	15
BP	collagen catabolic process	6.35	7.25E-09	8
BP	retinoic acid receptor signaling pathway	5.56	3.16E-09	7
BP	response to oxidative stress	7.94	1.33E-07	10
BP	cholesterol metabolic process	6.35	1.16E-06	8
BP	monoterpenoid metabolic process	3.17	8.95E-06	4
BP	steroid hormone receptor signaling pathway	3.97	2.17E-04	5
BP	regulation of bile acid biosynthetic process	2.38	4.06E-04	3
BP	cellular response to dexamethasone stimulus	3.17	9.31E-04	4
BP	glucose homeostasis	4.76	1.00E-03	6
CC	membrane raft	14.29	1.42E-14	18
CC	plasma membrane	54.76	5.45E-11	69
CC	cell surface	18.25	7.86E-11	23
CC	axon	11.11	3.50E-07	14
CC	endoplasmic reticulum membrane	18.25	2.20E-06	23
CC	neuronal cell body	10.32	3.07E-06	13
CC	perinuclear region of cytoplasm	14.29	3.18E-06	18
CC	caveola	5.56	6.21E-06	7
CC	RNA polymerase II transcription regulator complex	6.35	1.16E-05	8
CC	focal adhesion	10.32	1.22E-05	13
MF	nuclear receptor activity	11.90	2.97E-19	15
MF	peptidase activity	11.11	5.08E-14	14
MF	endopeptidase activity	10.32	6.22E-14	13
MF	enzyme binding	16.67	4.11E-13	21
MF	heme binding	11.90	1.70E-12	15
MF	zinc ion binding	21.43	9.71E-11	27
MF	serine-type endopeptidase activity	11.11	4.06E-10	14
MF	protein homodimerization activity	18.25	3.12E-09	23
MF	nuclear steroid receptor activity	5.56	6.21E-09	7
MF	identical protein binding	26.98	2.11E-08	34

Table S2. Pathways enriched in KEGG analysis

Description	Gene ratio	PValue	Count
Pathways in cancer	26.98412698	4.01E-14	34
Lipid and atherosclerosis	17.46031746	7.11E-13	22
AGE-RAGE signaling pathway in diabetic complications	9.523809524	9.20E-08	12
Arachidonic acid metabolism	7.936507937	1.34E-07	10
Fluid shear stress and atherosclerosis	10.31746032	3.63E-07	13
IL-17 signaling pathway	8.73015873	5.03E-07	11
TNF signaling pathway	8.73015873	4.04E-06	11
Thyroid hormone signaling pathway	8.73015873	5.06E-06	11
Endocrine resistance	7.936507937	6.56E-06	10
Adipocytokine signaling pathway	6.349206349	3.92E-05	8
PPAR signaling pathway	6.349206349	6.69E-05	8
Regulation of lipolysis in adipocytes	5.555555556	1.30E-04	7
Non-alcoholic fatty liver disease	7.936507937	2.50E-04	10
Bile secretion	5.555555556	0.001282	7
Apoptosis	6.349206349	0.002306	8

Table S3. Meta-regression effect size of blood lipids and moderator variables.

	coefficient	95%CI	t	P-value
TC				
Publication year	-0.891	-1.891,0.109	-1.85	0.078
N	0.080	-0.133,0.294	0.78	0.442
Region	-0.053	-0.932,0.827	-0.12	0.902
Blood lipid level	0.036	-0.123,0.194	0.47	0.645
Age	-0.057	-0.373,0.259	-0.38	0.710
BMI	0.243	-1.003,1.490	0.41	0.689
TG				
Publication year	1.014	-2.010,4.038	0.71	0.487
N	0.187	-0.458,0.831	0.61	0.548
Region	1.810	-0.471,4.091	1.68	0.112
Blood lipid level	0.192	-0.231,0.615	0.96	0.350
Age	-0.143	-0.959,0.672	-0.37	0.715
BMI	0.548	-3.079,4.175	0.32	0.753
HDL-C				
Publication year	0.140	-0.252,0.532	0.74	0.465
N	0.062	-0.029,0.154	1.42	0.172
Region	0.050	-0.267,0.368	0.33	0.746
Blood lipid level	-0.029	-0.090,0.032	-1.00	0.329
Age	0.032	-0.089,0.153	0.56	0.583
BMI	-0.267	-0.666,0.132	-1.40	0.178
LDL-C				
Publication year	-0.569	-1.595,0.458	-1.16	0.261
N	-0.032	-0.262,0.197	-0.29	0.773
Region	-0.222	-1.067,0.623	-0.55	0.590
Blood lipid level	-0.043	-0.189,0.103	-0.62	0.544
Age	-0.048	-0.359,0.262	-0.32	0.749
BMI	-0.012	-1.179,1.154	-0.02	0.983

Table S4. Dose–response analysis parameters.

	TC	TG	HDL-C	LDL-C
P-Values for linearity	0.8246	0.6664	0.2151	0.6270
P-Values for nonlinearity	0.5892	0.3711	0.0867	0.3426
